# Pyridine-2-carboximidamidate chloride monohydrate

**DOI:** 10.1107/S1600536810046106

**Published:** 2010-11-13

**Authors:** Qifan Chen, Huidong Zhang, Fang Zhang, Fei Liu

**Affiliations:** a Experiment Center, Eastern Liaoning University, No. 325 Wenhua Road, Yuanbao District, Dandong City, Liaoning Province 118003, People’s Republic of China; b College of Chemical Engineering & Materials, Eastern Liaoning University, No. 325 Wenhua Road, Yuanbao District, Dandong City, Liaoning Province 118003, People’s Republic of China

## Abstract

The title compound, C_6_H_8_N_3_^+^·Cl^−^·H_2_O, crystallizes with three formula units in the asymmetric unit. The cations are non-planar with the –C(NH_2_)_2_ groups twisted out of the ring planes. Each pyridine carboximidamidate cation is linked to another cation through N—H⋯N hydrogen bonds, to chloride ions by N—H⋯Cl hydrogen bonds, and to water mol­ecules by N—H⋯O hydrogen bonds. Water mol­ecules and chloride ions are also linked together *via* O—H⋯Cl hydrogen bonds. In the crystal, all these inter­molecular inter­actions result in a three-dimensional network.

## Related literature

For related structures, see: Guo *et al.* (2005 ▶); Fan *et al.* (2009 ▶); Góker *et al.* (2005 ▶); Walther *et al.* (2006 ▶).
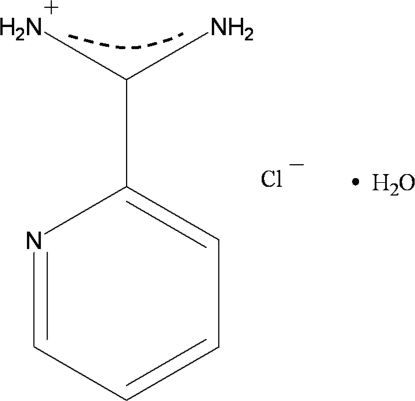


## Experimental

### 

#### Crystal data

C_6_H_8_N_3_^+^·Cl^−^·H_2_O
*M* *_r_* = 175.62
Triclinic, 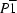

*a* = 7.2570 (15) Å
*b* = 11.146 (2) Å
*c* = 16.862 (3) Å
α = 79.24 (3)°
β = 82.14 (3)°
γ = 78.34 (3)°
*V* = 1305.3 (5) Å^3^
*Z* = 6
Mo *K*α radiation
μ = 0.39 mm^−1^
*T* = 293 K
0.25 × 0.24 × 0.22 mm

#### Data collection

Rigaku R-AXIS RAPID diffractometer
Absorption correction: multi-scan (*ABSCOR*; Higashi, 1995 ▶) *T*_min_ = 0.909, *T*_max_ = 0.918
12872 measured reflections
5919 independent reflections
4550 reflections with *I* > 2σ(*I*)
*R*_int_ = 0.024

#### Refinement

*R*[*F* ^2^ > 2σ(*F* ^2^)] = 0.035
*wR*(*F* ^2^) = 0.117
*S* = 1.11
5919 reflections
418 parameters
All H-atom parameters refined
Δρ_max_ = 0.23 e Å^−3^
Δρ_min_ = −0.26 e Å^−3^

### 

Data collection: *RAPID-AUTO* (Rigaku, 1998) ▶; cell refinement: *RAPID-AUTO*; data reduction: *RAPID-AUTO*; program(s) used to solve structure: *SHELXS97* (Sheldrick, 2008 ▶); program(s) used to refine structure: *SHELXL97* (Sheldrick, 2008 ▶); molecular graphics: *DIAMOND* (Brandenburg, 1999 ▶) and *SHELXP97* (Sheldrick, 2008 ▶); software used to prepare material for publication: *SHELXL97*.

## Supplementary Material

Crystal structure: contains datablocks global, I. DOI: 10.1107/S1600536810046106/zq2070sup1.cif

Structure factors: contains datablocks I. DOI: 10.1107/S1600536810046106/zq2070Isup2.hkl

Additional supplementary materials:  crystallographic information; 3D view; checkCIF report

## Figures and Tables

**Table 1:** Hydrogen-bond geometry (Å, °)

*D*—H⋯*A*	*D*—H	H⋯*A*	*D*⋯*A*	*D*—H⋯*A*
N23—H23*B*⋯O1^i^	0.87 (2)	1.97 (2)	2.829 (2)	173 (2)
N22—H22*B*⋯Cl1	0.89 (3)	2.31 (3)	3.187 (2)	169 (2)
N12—H12*B*⋯Cl3^ii^	0.91 (2)	2.35 (2)	3.2522 (19)	170.2 (18)
O1—H1*B*⋯Cl3	0.80 (3)	2.37 (3)	3.163 (2)	169 (2)
N2—H2*A*⋯Cl2^iii^	0.85 (3)	2.49 (3)	3.326 (2)	167 (2)
O1—H10⋯Cl2^iv^	0.98 (4)	2.22 (4)	3.1902 (19)	173 (3)
N3—H3*A*⋯O2	0.87 (2)	1.94 (2)	2.808 (2)	174.9 (19)
N2—H2*B*⋯Cl1	0.96 (2)	2.32 (2)	3.2754 (17)	170.9 (17)
N12—H12*A*⋯Cl3^v^	0.89 (2)	2.43 (2)	3.313 (2)	171 (2)
N23—H23*A*⋯Cl3^vi^	0.87 (2)	2.50 (2)	3.324 (2)	158 (2)
N13—H13*B*⋯O3^ii^	0.92 (2)	1.97 (2)	2.883 (2)	178 (2)
N22—H22*A*⋯Cl2^iii^	0.91 (2)	2.43 (3)	3.3194 (18)	168 (2)
N13—H13*A*⋯Cl1	0.91 (2)	2.48 (2)	3.335 (2)	156.9 (19)
O3—H03*B*⋯Cl2^iv^	0.84 (4)	2.45 (4)	3.286 (2)	174 (3)
O2—H0*B*⋯Cl1	0.85 (3)	2.28 (3)	3.1171 (18)	172 (2)
O2—H0*A*⋯Cl2	0.79 (3)	2.40 (3)	3.1833 (18)	175 (3)
O3—H03*A*⋯Cl3	0.94 (4)	2.25 (4)	3.1586 (18)	164 (3)

Symmetry codes: (i) 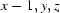; (ii) 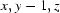; (iii) 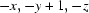; (iv) 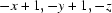; (v) 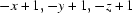; (vi) 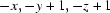.

**Fig. 1.**
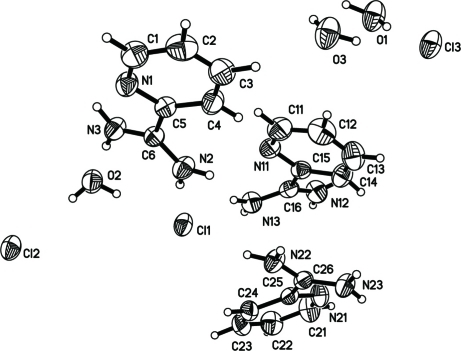
The molecular structure of the title compound showing 30% probability displacement ellipsoids for non-H atoms.

**Fig. 2.**
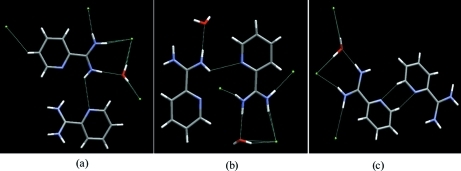
View of the hydrogen bonding interactions in the title compound.

**Fig. 3.**
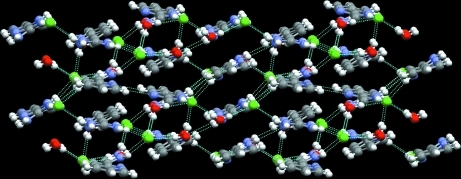
Crystal packing of the title compound.

## supplementary crystallographic information

### Comment

The pyridinecarboximidamidate derivatives have attracted a great attention due to their very potent antibacterial activities, interesting biological properties and applications in coordination chemistry, but a few samples have been reported (Guo *et al.,* 2005; Góker *et al.*, 2005; Walther *et al.*, 2006; Fan *et al.,* 2009). Here we report the structure of title compound C_6_H_8_N_3_^+^.Cl^-^.H_2_O. The title compound is an organic salt and crystallizes with three formula units in the asymmetric unit (Fig. 1). The molecules are nonplanar with the C(NH_2_)_2_ groups twisted out of the ring planes. The bond lengths and angles exhibit normal values and are comparable with those in the related structures (Guo *et al.,* 2005; Fan *et al.*, 2009).

In the crystal structure, pyridine carboximidamidate cations adopt three different connecting types (Fig. 2). Each pyridine carboximidamidate cation is linked to another cation through N—H···N hydrogen bonds, to chloride ions by N—H···Cl hydrogen bonds, and to water molecules by N—H···O hydrogen bonds. Water molecules and chloride ions are also linked together *via* O—H···Cl hydrogen bonds (Table 1). In the crystal structure, all these intermolecular interactions result in a three-dimensional network (Fig. 3).

### Experimental

To a solution of sodium methoxide (5.15 mmol) in methanol (50 mL) was added 2-cyanopyridine (5.2 g, 4.99 mmol). The mixture was stirred at room temperature for 4 h. Then ammonium chloride (2.9 g, 5.42 mmol) was slowly added to the resulting solution and the mixture was stirred at room temperature for 68 h. The resulting suspension was filtered and the solvent was removed from the filtrate under reduced pressure. Purification by wash with ethyl ether gave 2-amidinopyridine hydrochloride (5.62 g, 92%) as an off-white solid. Colorless crystals were formed after several days.

### Refinement

The H atoms were initially found in a difference Fourier map and their coordinates were refined freely.

### Crystal data

**Table:**

C_6_H_8_N_3_^+^·Cl^−^·H_2_O	*Z* = 6
*M**_r_* = 175.62	*F*(000) = 552
Triclinic, *P*1	*D*_x_ = 1.340 Mg m^−^^3^
Hall symbol: -P 1	Mo *K*α radiation, λ = 0.71073 Å
*a* = 7.2570 (15) Å	Cell parameters from 9445 reflections
*b* = 11.146 (2) Å	θ = 3.0–27.4°
*c* = 16.862 (3) Å	µ = 0.39 mm^−^^1^
α = 79.24 (3)°	*T* = 293 K
β = 82.14 (3)°	Block, colourless
γ = 78.34 (3)°	0.25 × 0.24 × 0.22 mm
*V* = 1305.3 (5) Å^3^	

### Data collection

**Table:**

Rigaku R-AXIS RAPID diffractometer	5919 independent reflections
Radiation source: fine-focus sealed tube	4550 reflections with *I* > 2σ(*I*)
graphite	*R*_int_ = 0.024
Detector resolution: 10 pixels mm^-1^	θ_max_ = 27.5°, θ_min_ = 3.0°
ω scans	*h* = −9→9
Absorption correction: multi-scan (*ABSCOR*; Higashi, 1995)	*k* = −14→14
*T*_min_ = 0.909, *T*_max_ = 0.918	*l* = −21→21
12872 measured reflections	

### Refinement

**Table:**

Refinement on *F*^2^	Primary atom site location: structure-invariant direct methods
Least-squares matrix: full	Secondary atom site location: difference Fourier map
*R*[*F*^2^ > 2σ(*F*^2^)] = 0.035	Hydrogen site location: inferred from neighbouring sites
*wR*(*F*^2^) = 0.117	All H-atom parameters refined
*S* = 1.11	*w* = 1/[σ^2^(*F*_o_^2^) + (0.0684*P*)^2^] where *P* = (*F*_o_^2^ + 2*F*_c_^2^)/3
5919 reflections	(Δ/σ)_max_ = 0.001
418 parameters	Δρ_max_ = 0.23 e Å^−^^3^
0 restraints	Δρ_min_ = −0.26 e Å^−^^3^

### Special details

**Table:**

Geometry. All e.s.d.'s (except the e.s.d. in the dihedral angle between two l.s. planes) are estimated using the full covariance matrix. The cell e.s.d.'s are taken into account individually in the estimation of e.s.d.'s in distances, angles and torsion angles; correlations between e.s.d.'s in cell parameters are only used when they are defined by crystal symmetry. An approximate (isotropic) treatment of cell e.s.d.'s is used for estimating e.s.d.'s involving l.s. planes.
Refinement. Refinement of *F*^2^ against ALL reflections. The weighted *R*-factor *wR* and goodness of fit *S* are based on *F*^2^, conventional *R*-factors *R* are based on *F*, with *F* set to zero for negative *F*^2^. The threshold expression of *F*^2^ > σ(*F*^2^) is used only for calculating *R*-factors(gt) *etc*. and is not relevant to the choice of reflections for refinement. *R*-factors based on *F*^2^ are statistically about twice as large as those based on *F*, and *R*- factors based on ALL data will be even larger.

### Fractional atomic coordinates and isotropic or equivalent isotropic displacement parameters (Å^2^)

**Table:**

	*x*	*y*	*z*	*U*_iso_*/*U*_eq_	
Cl1	0.17320 (7)	0.30230 (4)	0.10288 (2)	0.04346 (13)	
O1	0.7888 (3)	0.60439 (14)	0.40146 (10)	0.0647 (4)	
H1B	0.690 (4)	0.647 (3)	0.4141 (15)	0.073 (8)*	
H10	0.821 (5)	0.635 (3)	0.344 (2)	0.120 (11)*	
N1	0.3062 (2)	0.86483 (13)	−0.07194 (8)	0.0432 (3)	
C1	0.3052 (3)	0.98536 (18)	−0.07462 (13)	0.0540 (5)	
H1	0.336 (4)	1.031 (2)	−0.1243 (15)	0.078 (7)*	
Cl2	0.13596 (7)	0.30724 (4)	−0.21061 (3)	0.04888 (14)	
O2	0.3482 (2)	0.37193 (13)	−0.07519 (9)	0.0530 (4)	
H0B	0.305 (4)	0.345 (2)	−0.0273 (16)	0.077 (8)*	
H0A	0.297 (4)	0.351 (2)	−0.1073 (17)	0.075 (8)*	
N2	0.1754 (2)	0.60101 (14)	0.06004 (10)	0.0429 (3)	
H2A	0.111 (4)	0.629 (2)	0.1009 (15)	0.071 (7)*	
H2B	0.180 (3)	0.513 (2)	0.0663 (12)	0.053 (6)*	
C2	0.2604 (3)	1.04352 (17)	−0.00802 (14)	0.0561 (5)	
H2	0.261 (3)	1.131 (2)	−0.0152 (12)	0.055 (6)*	
Cl3	0.43514 (7)	0.80296 (4)	0.45146 (3)	0.05099 (14)	
O3	0.4557 (3)	0.86529 (14)	0.26022 (10)	0.0627 (4)	
H03B	0.561 (5)	0.826 (3)	0.2445 (19)	0.102 (11)*	
H03A	0.448 (5)	0.831 (3)	0.316 (2)	0.121 (12)*	
N3	0.3540 (2)	0.61884 (14)	−0.06282 (9)	0.0426 (3)	
H3B	0.410 (3)	0.669 (2)	−0.1007 (13)	0.054 (6)*	
H3A	0.350 (3)	0.544 (2)	−0.0696 (12)	0.051 (5)*	
C3	0.2174 (3)	0.97567 (18)	0.06587 (13)	0.0564 (5)	
H3	0.189 (3)	1.014 (2)	0.1127 (14)	0.070 (7)*	
C4	0.2181 (3)	0.85015 (18)	0.07107 (11)	0.0478 (4)	
H4	0.192 (3)	0.800 (2)	0.1214 (14)	0.067 (7)*	
C5	0.2600 (2)	0.80020 (14)	0.00027 (9)	0.0343 (3)	
C6	0.2627 (2)	0.66679 (14)	−0.00077 (9)	0.0338 (3)	
N11	0.4303 (2)	0.37190 (13)	0.25233 (9)	0.0428 (3)	
C11	0.4055 (3)	0.49449 (19)	0.24780 (13)	0.0540 (5)	
H11	0.446 (3)	0.542 (2)	0.2001 (13)	0.058 (6)*	
N12	0.4363 (3)	0.09814 (16)	0.39452 (10)	0.0502 (4)	
H12B	0.440 (3)	0.014 (2)	0.4040 (13)	0.055 (6)*	
H12A	0.456 (3)	0.128 (2)	0.4371 (15)	0.065 (7)*	
C12	0.3223 (3)	0.55573 (19)	0.31194 (14)	0.0604 (5)	
H12	0.311 (4)	0.648 (2)	0.3024 (15)	0.081 (8)*	
N13	0.3872 (2)	0.12940 (15)	0.25963 (10)	0.0447 (3)	
H13B	0.413 (3)	0.045 (2)	0.2600 (13)	0.059 (6)*	
H13A	0.360 (3)	0.187 (2)	0.2153 (13)	0.060 (6)*	
C13	0.2613 (3)	0.4879 (2)	0.38308 (14)	0.0599 (5)	
H13	0.207 (4)	0.528 (2)	0.4224 (16)	0.078 (8)*	
C14	0.2851 (3)	0.36072 (18)	0.38966 (12)	0.0477 (4)	
H14	0.241 (3)	0.3113 (19)	0.4350 (12)	0.048 (5)*	
C15	0.3718 (2)	0.30783 (15)	0.32274 (10)	0.0368 (3)	
C16	0.4002 (2)	0.17137 (16)	0.32577 (10)	0.0384 (4)	
N21	−0.0617 (2)	0.10605 (13)	0.39607 (8)	0.0441 (3)	
C21	−0.0357 (3)	−0.01675 (19)	0.40054 (13)	0.0563 (5)	
H21	−0.005 (3)	−0.059 (2)	0.4527 (13)	0.062 (6)*	
N22	−0.0909 (2)	0.38620 (15)	0.25737 (9)	0.0446 (3)	
H22B	−0.032 (3)	0.359 (2)	0.2128 (15)	0.066 (7)*	
H22A	−0.111 (3)	0.470 (2)	0.2526 (14)	0.069 (7)*	
C22	−0.0480 (3)	−0.07293 (19)	0.33556 (13)	0.0559 (5)	
H22	−0.030 (3)	−0.160 (2)	0.3423 (13)	0.061 (6)*	
N23	−0.2026 (2)	0.35240 (16)	0.39229 (10)	0.0453 (4)	
H23B	−0.215 (3)	0.430 (2)	0.3963 (13)	0.058 (6)*	
H23A	−0.232 (3)	0.299 (2)	0.4342 (14)	0.057 (6)*	
C23	−0.0891 (3)	−0.00047 (19)	0.26314 (12)	0.0526 (5)	
H23	−0.103 (4)	−0.035 (2)	0.2163 (15)	0.077 (7)*	
C24	−0.1160 (3)	0.12748 (18)	0.25641 (10)	0.0431 (4)	
H24	−0.146 (3)	0.1764 (18)	0.2077 (12)	0.048 (5)*	
C25	−0.1009 (2)	0.17539 (15)	0.32452 (9)	0.0338 (3)	
C26	−0.1327 (2)	0.31196 (15)	0.32427 (9)	0.0352 (3)	

### Atomic displacement parameters (Å^2^)

**Table:**

	*U*^11^	*U*^22^	*U*^33^	*U*^12^	*U*^13^	*U*^23^
Cl1	0.0579 (3)	0.0378 (2)	0.0333 (2)	−0.01169 (18)	0.00298 (17)	−0.00477 (16)
O1	0.0845 (12)	0.0469 (8)	0.0521 (9)	0.0040 (8)	0.0066 (8)	−0.0091 (7)
N1	0.0551 (9)	0.0372 (8)	0.0361 (7)	−0.0117 (7)	−0.0026 (6)	−0.0009 (6)
C1	0.0739 (14)	0.0379 (10)	0.0502 (11)	−0.0197 (9)	−0.0090 (10)	0.0050 (9)
Cl2	0.0640 (3)	0.0430 (2)	0.0393 (2)	−0.0127 (2)	0.00567 (19)	−0.01046 (18)
O2	0.0740 (10)	0.0492 (8)	0.0412 (7)	−0.0272 (7)	0.0026 (7)	−0.0098 (6)
N2	0.0535 (9)	0.0310 (7)	0.0428 (8)	−0.0122 (7)	0.0073 (7)	−0.0061 (6)
C2	0.0651 (13)	0.0295 (9)	0.0749 (14)	−0.0119 (8)	−0.0056 (11)	−0.0094 (9)
Cl3	0.0642 (3)	0.0439 (2)	0.0370 (2)	−0.0035 (2)	0.00486 (19)	−0.00088 (18)
O3	0.0851 (12)	0.0472 (8)	0.0474 (8)	−0.0042 (8)	0.0026 (8)	−0.0020 (7)
N3	0.0529 (9)	0.0337 (8)	0.0407 (8)	−0.0116 (7)	0.0084 (7)	−0.0102 (7)
C3	0.0654 (13)	0.0476 (11)	0.0625 (12)	−0.0181 (10)	0.0094 (10)	−0.0280 (10)
C4	0.0608 (12)	0.0435 (10)	0.0397 (9)	−0.0164 (9)	0.0071 (8)	−0.0098 (8)
C5	0.0341 (8)	0.0301 (8)	0.0375 (8)	−0.0047 (6)	−0.0016 (6)	−0.0056 (6)
C6	0.0351 (8)	0.0304 (8)	0.0346 (8)	−0.0056 (6)	−0.0029 (6)	−0.0032 (6)
N11	0.0463 (8)	0.0403 (8)	0.0413 (8)	−0.0133 (7)	−0.0022 (6)	−0.0011 (6)
C11	0.0637 (13)	0.0431 (10)	0.0535 (11)	−0.0168 (9)	−0.0050 (10)	0.0036 (9)
N12	0.0662 (11)	0.0378 (9)	0.0454 (9)	−0.0115 (8)	−0.0133 (8)	0.0036 (7)
C12	0.0682 (14)	0.0369 (10)	0.0749 (15)	−0.0044 (9)	−0.0132 (11)	−0.0077 (10)
N13	0.0533 (9)	0.0357 (8)	0.0439 (8)	−0.0081 (7)	−0.0068 (7)	−0.0020 (7)
C13	0.0672 (14)	0.0520 (12)	0.0582 (12)	0.0050 (10)	−0.0078 (11)	−0.0191 (10)
C14	0.0487 (10)	0.0482 (10)	0.0398 (9)	−0.0024 (8)	−0.0011 (8)	−0.0003 (8)
C15	0.0332 (8)	0.0374 (9)	0.0389 (8)	−0.0072 (7)	−0.0064 (7)	−0.0013 (7)
C16	0.0322 (8)	0.0416 (9)	0.0394 (8)	−0.0090 (7)	−0.0025 (7)	0.0000 (7)
N21	0.0555 (9)	0.0392 (8)	0.0350 (7)	−0.0006 (7)	−0.0085 (6)	−0.0053 (6)
C21	0.0746 (14)	0.0421 (10)	0.0474 (10)	0.0028 (9)	−0.0149 (10)	−0.0041 (8)
N22	0.0531 (9)	0.0370 (8)	0.0395 (8)	−0.0089 (7)	0.0081 (7)	−0.0044 (7)
C22	0.0681 (14)	0.0391 (10)	0.0615 (12)	−0.0083 (9)	−0.0054 (10)	−0.0137 (9)
N23	0.0547 (10)	0.0402 (9)	0.0387 (8)	−0.0099 (7)	0.0087 (7)	−0.0088 (7)
C23	0.0634 (13)	0.0548 (11)	0.0470 (10)	−0.0201 (10)	−0.0012 (9)	−0.0202 (9)
C24	0.0499 (10)	0.0495 (10)	0.0328 (8)	−0.0184 (8)	−0.0036 (7)	−0.0046 (7)
C25	0.0287 (7)	0.0381 (8)	0.0337 (7)	−0.0066 (6)	0.0006 (6)	−0.0060 (6)
C26	0.0309 (8)	0.0383 (9)	0.0355 (8)	−0.0074 (7)	0.0001 (6)	−0.0053 (7)

### Geometric parameters (Å, °)

**Table:**

O1—H1B	0.80 (3)	N12—H12A	0.89 (2)
O1—H10	0.98 (4)	C12—C13	1.363 (3)
N1—C5	1.331 (2)	C12—H12	1.00 (3)
N1—C1	1.334 (2)	N13—C16	1.309 (2)
C1—C2	1.369 (3)	N13—H13B	0.92 (2)
C1—H1	0.92 (2)	N13—H13A	0.91 (2)
O2—H0B	0.85 (3)	C13—C14	1.378 (3)
O2—H0A	0.79 (3)	C13—H13	0.87 (3)
N2—C6	1.311 (2)	C14—C15	1.386 (3)
N2—H2A	0.85 (3)	C14—H14	0.91 (2)
N2—H2B	0.96 (2)	C15—C16	1.485 (2)
C2—C3	1.364 (3)	N21—C21	1.333 (2)
C2—H2	0.96 (2)	N21—C25	1.338 (2)
O3—H03B	0.84 (4)	C21—C22	1.381 (3)
O3—H03A	0.94 (4)	C21—H21	0.95 (2)
N3—C6	1.302 (2)	N22—C26	1.307 (2)
N3—H3B	0.88 (2)	N22—H22B	0.89 (3)
N3—H3A	0.87 (2)	N22—H22A	0.91 (2)
C3—C4	1.384 (3)	C22—C23	1.366 (3)
C3—H3	0.95 (2)	C22—H22	0.94 (2)
C4—C5	1.381 (2)	N23—C26	1.311 (2)
C4—H4	0.94 (2)	N23—H23B	0.87 (2)
C5—C6	1.486 (2)	N23—H23A	0.87 (2)
N11—C15	1.330 (2)	C23—C24	1.385 (3)
N11—C11	1.331 (2)	C23—H23	0.97 (2)
C11—C12	1.390 (3)	C24—C25	1.380 (2)
C11—H11	0.92 (2)	C24—H24	0.924 (19)
N12—C16	1.312 (2)	C25—C26	1.492 (2)
N12—H12B	0.91 (2)		
			
H1B—O1—H10	106 (3)	C16—N13—H13A	116.6 (13)
C5—N1—C1	116.70 (15)	H13B—N13—H13A	124.4 (19)
N1—C1—C2	123.59 (18)	C12—C13—C14	119.3 (2)
N1—C1—H1	117.3 (16)	C12—C13—H13	117.7 (17)
C2—C1—H1	119.1 (16)	C14—C13—H13	123.0 (17)
H0B—O2—H0A	111 (2)	C13—C14—C15	117.69 (19)
C6—N2—H2A	125.2 (16)	C13—C14—H14	122.7 (13)
C6—N2—H2B	124.9 (13)	C15—C14—H14	119.5 (12)
H2A—N2—H2B	110 (2)	N11—C15—C14	124.30 (16)
C3—C2—C1	119.11 (17)	N11—C15—C16	115.39 (15)
C3—C2—H2	122.4 (12)	C14—C15—C16	120.29 (16)
C1—C2—H2	118.5 (12)	N13—C16—N12	122.82 (17)
H03B—O3—H03A	100 (3)	N13—C16—C15	118.41 (15)
C6—N3—H3B	116.3 (14)	N12—C16—C15	118.77 (16)
C6—N3—H3A	122.4 (14)	C21—N21—C25	116.99 (15)
H3B—N3—H3A	120.9 (19)	N21—C21—C22	122.95 (18)
C2—C3—C4	118.91 (18)	N21—C21—H21	111.6 (13)
C2—C3—H3	120.1 (14)	C22—C21—H21	125.4 (13)
C4—C3—H3	121.0 (14)	C26—N22—H22B	123.0 (15)
C5—C4—C3	117.91 (18)	C26—N22—H22A	124.0 (15)
C5—C4—H4	121.0 (13)	H22B—N22—H22A	113 (2)
C3—C4—H4	121.1 (13)	C23—C22—C21	119.16 (18)
N1—C5—C4	123.74 (15)	C23—C22—H22	121.3 (13)
N1—C5—C6	114.14 (13)	C21—C22—H22	119.5 (13)
C4—C5—C6	122.09 (15)	C26—N23—H23B	121.2 (15)
N3—C6—N2	122.05 (15)	C26—N23—H23A	118.2 (14)
N3—C6—C5	118.14 (15)	H23B—N23—H23A	121 (2)
N2—C6—C5	119.81 (15)	C22—C23—C24	119.30 (17)
C15—N11—C11	116.63 (16)	C22—C23—H23	122.4 (15)
N11—C11—C12	123.22 (19)	C24—C23—H23	118.3 (15)
N11—C11—H11	119.1 (14)	C25—C24—C23	117.51 (17)
C12—C11—H11	117.7 (14)	C25—C24—H24	123.3 (12)
C16—N12—H12B	124.8 (13)	C23—C24—H24	119.2 (12)
C16—N12—H12A	121.0 (15)	N21—C25—C24	124.10 (15)
H12B—N12—H12A	114.1 (19)	N21—C25—C26	114.20 (14)
C13—C12—C11	118.88 (19)	C24—C25—C26	121.68 (15)
C13—C12—H12	124.1 (15)	N22—C26—N23	122.63 (16)
C11—C12—H12	117.0 (15)	N22—C26—C25	119.59 (15)
C16—N13—H13B	119.0 (13)	N23—C26—C25	117.78 (15)

### Hydrogen-bond geometry (Å, °)

**Table:**

*D*—H···*A*	*D*—H	H···*A*	*D*···*A*	*D*—H···*A*
N23—H23B···O1^i^	0.87 (2)	1.97 (2)	2.829 (2)	173 (2)
N22—H22B···Cl1	0.89 (3)	2.31 (3)	3.187 (2)	169 (2)
N12—H12B···Cl3^ii^	0.91 (2)	2.35 (2)	3.2522 (19)	170.2 (18)
O1—H1B···Cl3	0.80 (3)	2.37 (3)	3.163 (2)	169 (2)
N2—H2A···Cl2^iii^	0.85 (3)	2.49 (3)	3.326 (2)	167 (2)
O1—H10···Cl2^iv^	0.98 (4)	2.22 (4)	3.1902 (19)	173 (3)
N3—H3A···O2	0.87 (2)	1.94 (2)	2.808 (2)	174.9 (19)
N2—H2B···Cl1	0.96 (2)	2.32 (2)	3.2754 (17)	170.9 (17)
N12—H12A···Cl3^v^	0.89 (2)	2.43 (2)	3.313 (2)	171 (2)
N23—H23A···Cl3^vi^	0.87 (2)	2.50 (2)	3.324 (2)	158 (2)
N13—H13B···O3^ii^	0.92 (2)	1.97 (2)	2.883 (2)	178 (2)
N22—H22A···Cl2^iii^	0.91 (2)	2.43 (3)	3.3194 (18)	168 (2)
N13—H13A···Cl1	0.91 (2)	2.48 (2)	3.335 (2)	156.9 (19)
O3—H03B···Cl2^iv^	0.84 (4)	2.45 (4)	3.286 (2)	174 (3)
O2—H0B···Cl1	0.85 (3)	2.28 (3)	3.1171 (18)	172 (2)
O2—H0A···Cl2	0.79 (3)	2.40 (3)	3.1833 (18)	175 (3)
O3—H03A···Cl3	0.94 (4)	2.25 (4)	3.1586 (18)	164 (3)

Symmetry codes: (i) *x*−1, *y*, *z*; (ii) *x*, *y*−1, *z*; (iii) −*x*, −*y*+1, −*z*; (iv) −*x*+1, −*y*+1, −*z*; (v) −*x*+1, −*y*+1, −*z*+1; (vi) −*x*, −*y*+1, −*z*+1.
